# Parasitic Helminth Infections and Intron Sequence Genotyping of *Opisthorchis viverrini*-like Eggs in Outdoor Domestic Cats and Dogs Across the Chi River Basin, Maha Sarakham Province, Thailand

**DOI:** 10.3390/ijms26073005

**Published:** 2025-03-26

**Authors:** Kotchaphon Vaisusuk, Wasupon Chatan, Warayutt Pilap, Tongjit Thanchomnang, Chavanut Jaroenchaiwattanachote, Paiboon Sithithaworn, Ross H. Andrews, Chairat Tantrawatpan, Weerachai Saijuntha

**Affiliations:** 1 Department of Veterinary Technology and Veterinary Nursing, Faculty of Agricultural Technology, Rajabhat Maha Sarakham University, Maha Sarakham 44000, Thailand; kotchaphon@rmu.ac.th; 2 Department of Veterinary Clinic, Faculty of Veterinary Sciences, Mahasarakham University, Maha Sarakham 44000, Thailand; wasupon.c@msu.ac.th; 3 Center of Excellence in Biodiversity Research, Mahasarakham University, Maha Sarakham 44150, Thailand; warayutt@msu.ac.th (W.P.); chavanut.j@msu.ac.th (C.J.); 4 Walai Rukhavej Botanical Research Insitute, Mahasarakham University, Maha Sarakham 44150, Thailand; 5 Biomedical Science Research Unit, Faculty of Medicine, Mahasarakham University, Maha Sarakham 44000, Thailand; tongjit.t@msu.ac.th; 6 Department of Parasitology, Faculty of Medicine, Khon Kaen University, Khon Kaen 40002, Thailand; paib_sit@kku.ac.th; 7 Department of Surgery & Cancer, Faculty of Medicine, Imperial College, London SW7 2AZ, UK; rhandrews@imperial.ac.uk; 8 Division of Cell Biology, Department of Preclinical Sciences, Faculty of Medicine, Center of Excellence in Stem Cell Research and Innovation, Thammasat University, Rangsit Campus, Pathum Thani 12120, Thailand

**Keywords:** feline, canine, helminths, zoonoses, intron, genetic variation, molecular marker, *Opisthorchis viverrini*

## Abstract

This study investigates the prevalence of parasitic helminths in free-ranging domestic cats and dogs near the Chi River and natural reservoirs in Maha Sarakham Province, Thailand. Fecal samples from 39 cats and 148 dogs were analyzed using a modified formalin-ether concentration technique (FECT). The overall prevalence of helminth infections was 64.1% in cats and 51.4% in dogs. Common parasites were detected including soil-transmitted species like *Ancylostoma* sp. (hookworm), *Toxocara* spp., and *Strongyloides* sp., as well as foodborne helminths such as *Taenia* sp., *Hymenolepis* sp., *Spirometra* sp., and *Opisthorchis* sp. Multiple parasitic infections were commonly found in dogs (57.9%) and cats (46.2%). Our findings suggest that domestic cats and dogs act as important reservoirs for zoonotic helminths in the region. Notably, *Opisthorchis viverrini*-like eggs were found exclusively in cats, with a prevalence of 23.1%. The intron 5 of domain 1 of the taurocyamine kinase gene (TkD1Int5) was used for genotyping *O. viverrini*-like eggs. All *O. viverrini*-like egg samples with TkD1Int5 haplotypes (Ov116–Ov123) were uniquely found in cats. Genetic analysis revealed that TkD1Int5 haplotypes were similar to those previously reported for *Opisthorchis viverrini* in various species of cyprinid fish across opisthorchiasis-endemic regions in Thailand and Lao PDR. Three TkD1Int5 haplogroups (I–III) were classified, with *O. viverrini*-like eggs from cats distributed across all haplogroups. Notably, one haplotype (Ov118) was genetically distinct from the others and did not cluster into any haplogroup. These findings highlight the crucial role of cats as reservoir hosts and their potential contribution to the transmission of the zoonotic liver fluke *O. viverrini*, posing a notable public health concern.

## 1. Introduction

Domestic cats and dogs have been part of human society for over 10,000 years. The growing populations of domestic cats and dogs significantly contribute to disease transmission, particularly zoonotic helminths [1]. In rural areas of Thailand, free-ranging domestic cats and dogs face an increased risk of soil- and foodborne parasitic helminth infections. These infections can adversely affect animal health, leading to morbidity and mortality, especially in young and immunosuppressed animals. Cats and dogs can harbor a wide range of parasitic helminths, some of which are zoonotic and capable of causing severe diseases in humans [1]. While human hygienic practices may help disrupt the helminth life cycle, cats and dogs do not adhere to such practices. They contribute to the persistence of parasitic helminths by shedding eggs or larvae into the environment through their feces. These infective stages can contaminate soil and water, subsequently infecting intermediate hosts, which commonly are aquatic animals and plants. Mammalian final hosts, including humans, can become infected by consuming these second intermediate hosts, particularly when consumed raw or undercooked [2].

A high prevalence of soil-transmitted zoonotic parasites, such as hookworms, *Trichuris* sp., and *Toxocara* spp., has been widely reported in cats and dogs in Thailand. Additionally, foodborne helminths, such as *Spirometra* sp. and *Opisthorchis* sp., are also prevalent [3,4,5,6,7,8,9]. Three major river basins, namely the Mun, Chi, and Mekong are located in the Lower Mekong Subregion of northeastern Thailand. The Chi River Basin is a key endemic area for various parasitic helminths, particularly the carcinogenic liver fluke *Opisthorchis viverrini*. This basin covers approximately 49,480 km^2^ of flood-prone areas in northeastern Thailand [10]. Maha Sarakham Province, situated within the Chi River Basin, has a high prevalence of human opisthorchiasis [2]. In Thailand, particularly in the northeastern region, *O. viverrini* infection is widespread and strongly associated with the development of bile duct cancer (cholangiocarcinoma, CCA) [11]. In endemic areas, cats and dogs play a crucial role as reservoir hosts of this liver fluke [11]. Several studies have reported a high prevalence of *O. viverrini* infection in cats and dogs across various locations in the Lower Mekong Subregion, including the Chi River Basin [2,7,8,9,12].

In addition to *O. viverrini*, other species of liver flukes within the genus *Opisthorchis* have been reported in Southeast Asia. For example, *Opisthorchis lobatus* (Bilqees, Shabbir, & Parveen, 2003) was recently identified in freshwater fish in Lao PDR and may be zoonotic; however, its role in human infections remains unknown [13]. Several avian *Opisthorchis* species, including *Opisthorchis cheelis* Lal, 1939, *Opisthorchis longissimus* Stiles & Hassall, 1896, and *Opisthorchis parageminus* Oshmarin, 1970, have also been documented in Southeast Asia [14,15]. Additionally, an *O. viverrini*-like fluke was discovered in duck co-endemic with human *O. viverrini* [16]. Currently, the eggs of these liver flukes cannot be reliably distinguished at the species level. As a result, *O. viverrini*-like eggs found in mammalian hosts may not necessarily belong to *O. viverrini*. Mitochondrial DNA genotyping of *O. viverrini* recovered from humans and cats in an endemic area of Thailand initially suggested no distinct host specificity between the ‘human’ and ‘cat’ genotypes [17]. However, a more recent study provides updated insights into the genetic differences between *O. viverrini*-infected cats and humans. This report indicates that *O. viverrini*-like eggs found in cats are genetically distinct from those found in humans and are more closely related to *O. viverrini*-like metacercariae identified in snakehead fish *Channa striata* (Bloch, 1793). These findings suggest that snakehead fish may serve as a significant second intermediate host for the cat-specific genotype *O. viverrini* [18].

Currently, there is limited information on parasitic helminth infections in cats and dogs along the Chi River Basin in Maha Sarakham Province. We hypothesize that cats and dogs living near natural water sources are at high risk of parasitic helminth infection, particularly from the fish-borne zoonotic trematode *O. viverrini*. Several studies have suggested that intron sequences of taurocyamine kinase (*TK*) provide suitable resolution for identifying and differentiating trematode species [19,20]. More recently, intron 5 of domain 1 of *TK* (TkD1Int5) in *O. viverrini* and *Clonorchis sinensis* has been characterized and proposed as a potential genetic marker for examining intraspecific genetic variation [21]. Therefore, this study was conducted to investigate the prevalence of parasitic helminth infections in outdoor domestic cats and dogs living along the Chi River and nearby natural reservoirs in Maha Sarakham Province. *O. viverrini*-like eggs identified in this study will be genotyped using TkD1Int5 region as a molecular marker.

## 2. Results

### 2.1. Prevalence of Helminth Infection

**Figure 1:**
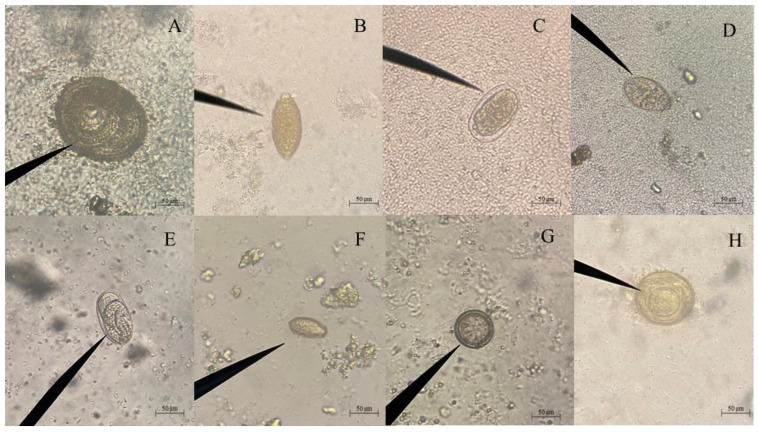
The parasite eggs found in the feces of cats and dogs under 400× magnification are examined using the modified formalin-ether concentration technique (FECT). (**A**) *Toxocara* spp.; (**B**) *Trichuris* sp.; (**C**) *Ancylostoma* sp.; (**D**) *Spirometra* sp.; (**E**) *Strongyloiodes* sp.; (**F**) *Opisthorchis* sp.; (**G**) *Taenia* sp.; and (**H**) *Hymenolepis* sp.

The prevalence of parasitic helminth infection found in cats and dogs was 64.1% and 51.4%, respectively (Table 1). Of the 76 infected dog fecal samples, 7 different species of gastrointestinal parasites were found, namely *Ancylostoma* sp. or hookworm (37.2%; 55/148), *Strongyloides* sp. (8.8%; 13/148), *Toxocara* spp. (22.3%; 33/148), *Trichuris* sp. (2.0%; 3/148), *Taenia* sp. (10.8%; 16/148), *Hymenolepis* sp. (2.0%; 3/148)*,* and *Spirometra* sp. (11.5%; 17/148) (Table 2). Of the 25 infected cat fecal samples, 5 different species of gastrointestinal parasites were detected, i.e., hookworms (46.2%; 18/39), *Strongyloides* sp. (15.4%; 6/39), *Toxocara* spp. (30.8%; 12/39), *Hymenolepis* sp. (2.6%; 1/39), and *Spirometra* sp. (5.1%; 2/39). Interestingly, in this study, *Opisthorchis*-like eggs were found in only cats, with a high prevalence of 23.1% (9/39) observed (Table 2). Photographs of all parasite eggs were taken and are shown in Figure 1.

Of the 76 infected dogs, multiple parasitic infections were detected in 44 dogs (57.9%) (Table 3). Among these, 27 dogs were co-infected with two parasites, 13 dogs with three parasites, 3 dogs with four parasites, and 1 dog with five parasites. The most prevalent co-infection was *Ancylostoma* sp. + *Toxocara* sp., observed in eight dogs (10.5%). Other notable mixed infections included *Ancylostoma* sp. + *Taenia* sp. in six dogs (7.9%), *Ancylostoma* sp. + *Strongyloides* sp. in five dogs (6.6%), *Ancylostoma* sp. + *Toxocara* sp. + *Spirometra* sp. in five dogs (6.6%), and *Ancylostoma* sp. + *Toxocara* sp. + *Strongyloides* sp. in four dogs (5.3%) (Table 3).

Of the 25 infected cats, multiple parasitic infections were detected in 18 cats (72%) (Table 4). Among these, 13 cats were co-infected with two parasites and 5 cats with three parasites. The most prevalent co-infection was *Ancylostoma* sp. + *Toxocara* sp., observed in seven cats (28%). Other notable mixed infections involving *Ophisthorchis* sp. and other parasites, including *Ancylostoma* sp., *Strongyloides* sp., *Spirometra* sp., and *Toxocara* sp., were also observed (Table 4).

### 2.2. Molecular Genotyping of O. viverrini

DNA was extracted from each of nine cat fecal samples that were *O. viverrini*-like egg positive and were subjected to PCR for TkD1Int5 amplification, but only five samples (Cat1–Cat5) were successfully amplified. TkD1Int5 sequences of *O. viverrini*-like eggs were deposited in GenBank under accession numbers PQ816749–PQ816756. TkD1Int5 sequencing revealed that the *O. viverrini*-like eggs found in five cats comprised eight unique haplotypes, designated Ov116 to Ov123. A mix of five haplotypes (Ov116–Ov120) was observed in cat number 2 (Cat2), while the other four cats (Cat1, Cat3–Cat5) found that only one haplotype infected each of them. Molecular diversity indices indicated high haplotype diversity, ranging from 0.618 ± 0.164 to 1.000 ± 0.045 (average 0.968 ± 0.007), with a large number of unique haplotypes identified in each locality, including those isolated from cats in this study (Table 5).

### 2.3. Haplotype Network and Phylogenetic Tree Analyses

**Figure 2:**
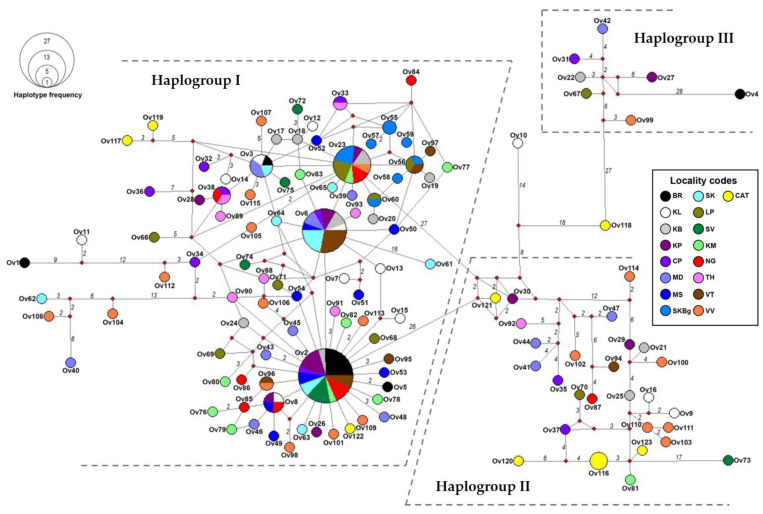
A minimum spanning haplotype network of the sequences of TkD1Int5 of *Opisthorchis viverrini* from various localities (Ov1–Ov115) and *O. viverrini*-like eggs from cats represented by the yellow circles (Ov1–Ov123). The area of the circles represents the proportion of the sample number found in each haplotype. The number in each branch represents mutational steps, whereas one mutational step is indicated without numbering. Locality codes are provided in Table 5.

The 185 TkD1Int5 sequences of *O. viverrini* from a previous study [21] together with 10 sequences of *O. viverrini*-like eggs generated in this study were used to construct a haplotype network. This network separated the sequences into three distinct haplogroups (I–III) based on mutational steps (ms) greater than 20. Haplogroup I is the largest group, containing haplotypes of *O. viverrini* from various localities, including three haplotypes of *O. viverrini*-like eggs, namely Ov117, Ov119, and Ov122. Haplotypes Ov116 and Ov120–Ov123 of *O. viverrini*-like eggs were grouped in haplogroup II. No haplotype of *O. viverrini*-like eggs was found to belong to haplogroup III (Figure 2). Interestingly, the Ov118 haplotype from Cat2 and the Ov10 haplotype from a cyprinid fish in Khon Kaen Province were genetically distinct from the others, with more than 20 mutational steps. These haplotypes did not cluster within any of the haplogroups identified in this study (Figure 2).

**Figure 3:**
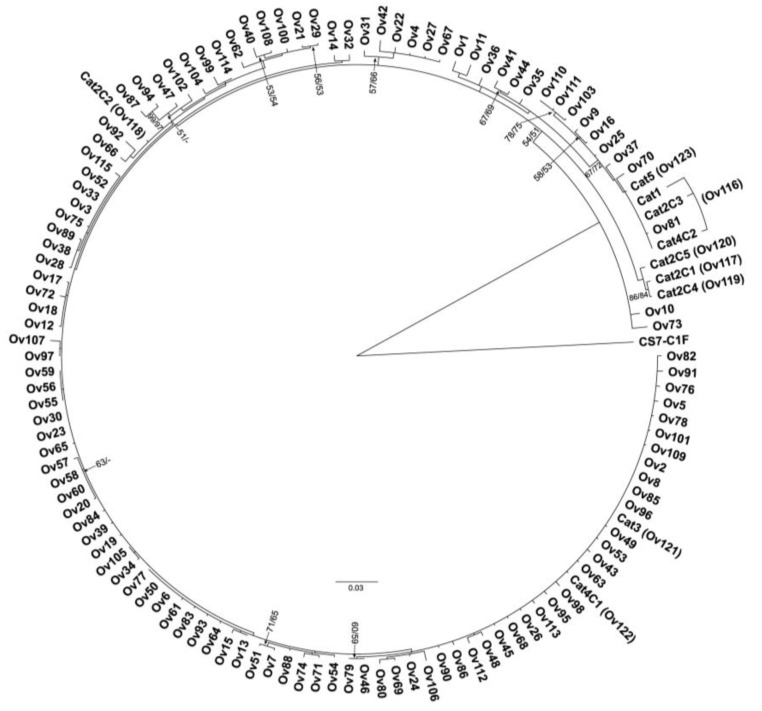
Phylogenetic tree constructed based on TkD1Int5 haplotypes of *Opisthorchis viverrini* TkD1Int5 sequences from various localities and *O. viverrini-*like eggs from cats (in parentheses). Nodal supports are the bootstrap values generated by neighbor-joining followed by those from maximum likelihood analyses.

A phylogenetic tree constructed using TkD1Int5 haplotypes of *O. viverrini* and *O. viverrini*-like eggs, with *Clonorchis sinensis* as the outgroup, showed that *O. viverrini*-like eggs from cats in this study formed a monophyletic group with *O. viverrini* from cyprinid fish from various localities in Thailand and Lao PDR. However, there appears to be evidence of a cat-specific genotype group, including haplotypes Ov117, Ov119, and Ov120 (Figure 3).

## 3. Discussion

The prevalence of parasitic helminths can vary due to many factors, such as geographical region, presence of veterinary care, habits of the local animal populations, season of the year, and population composition. In this study, we hypothesized that outdoor free-ranged domestic cats and dogs close to natural water sources could be a source of zoonotic infection. In Thailand, the prevalence of parasitic helminth infections in stray cats and dogs is very high [3,4,9]. The results of our study found a high prevalence of parasitic helminth infection in outdoor cats and dogs in the villages along the Chi River Basin or nearby natural water sources in Maha Sarakham Province, Thailand. This finding supports our hypothesis that outdoor cats and dogs are at high risk of infection by soil- and foodborne parasitic helminths.

In this study, the prevalence of intestinal parasites found was relatively high. Our results show that parasitic helminth infection in cats (64.1%) is higher than in dogs (51.4%). The most prevalent infections in cats and dogs are soil-transmission helminths, i.e., *Ancylostoma* sp., *Toxocara* spp., and *Strongyloides* sp. This finding is in concordance with that found in several previous reports. For example, a high prevalence of *Ancylostoma* sp. and *Toxocara* spp. infection in dogs and cats that were free-ranged outdoors has been reported in Bangkok [3,4]. Unsurprisingly, these pets have a high prevalence of soil-transmitted helminth infection. Furthermore, other countries also found a high prevalence of parasitic helminths in dogs, such as the 57.4% reported in a study conducted in Italy [22] and the 58.5% and 78% reported in Brazil and Mexico, respectively [23,24].

Several zoonotic nematodes have been found in this study; one important pathogenic is *Strongyloides* sp. was identified in 15.4% of domestic cats in our study. In a similar study conducted in the northeastern region of Brazil, the prevalence of intestinal parasites in household cats was 21.23% [25]. There is evidence of zoonotic transmission between humans and dogs, as determined by CO1 genotyping [26]. This result provides important information regarding the presence of *Strongyloides* sp., which may widely spread in the environment and, hence, increase the risk of human strongyloidiasis. The other three nematodes detected in our study are also potentially zoonotic, namely *Toxocara* spp., *Ancylostoma* sp. (hookworm), and *Trichuris* sp. Hookworm infections with *Ancylostoma caninum* (Ercolani, 1859) tend to be variable and focused in regional geographic areas. While the dominant species in the temperate climate zone is the genus *Toxocara*, hookworms are the most common intestinal parasite detected in the population of dogs and cats in tropical and subtropical climate zones [27].

One of the most common behaviors of cats and dogs is hunting for small mammals, amphibians, reptiles, rodents, and birds [1,28], including fish when they are free-ranged close to water sources. Thus, foodborne parasitic helminths can be transmitted via this prey behavior. We found infection of several foodborne helminths, namely *Taenia* sp., *Spirometra* sp., and *Opisthorchis* sp., in cats and/or dogs. Parasitic helminths detected in cats and dogs in this study are important zoonotic and pathogenic helminths, especially the carcinogenic liver fluke *O. viverrini*.

Dogs usually have a much lower prevalence of *O. viverrini* infection than cats [7,8]. However, some reports have the opposite [9], for instance, dogs were positive for *O. viverrini*-like eggs higher than cats. Dogs were not found to be infected with *O. viverrini*-like parasites in this study, which could be due to several factors. Limited exposure to infected fish, the secondary intermediate host, may reduce the likelihood of infection in dogs. Additionally, host specificity might play a role, as dogs may not serve as suitable hosts for these *O. viverrini*-like genotypes. The sample size of examined dogs could also have been insufficient to detect infections. Further studies with larger sample sizes and molecular analyses are needed to clarify the role of dogs in the transmission dynamics of *O. viverrini*-like parasites.

TkD1Int5 genotyping herein revealed that *O. viverrini*-like eggs in a cat were genetically similar to *O. viverrini* from previous reports, which was an *O. viverrini* population from naturally infected cyprinid fish [21]. Our finding supports an earlier study that the host specificity of the ‘human’ and ‘cat’ genotypes is not distinct [17]. A recent study reveals that *O. viverrini*-like eggs in cats are genetically distinct from those in humans and closely related to metacercariae in snakehead fish, suggesting the fish may serve as a key second intermediate host for cat-specific genotypes [18].

We found that one out of five cats were mixed-infected by five different haplotypes. This suggests multiple sources of infection or high genetic diversity of *O. viverrini* in the environment. Cats are known to roam over long distances [29], especially when hunting, which may expose them to infected intermediate hosts from various locations. This mobility could contribute to the acquisition of diverse parasite haplotypes and play a role in the dispersal of *O. viverrini*. However, future genotyping of TkD1Int5 in a larger sample size of *O. viverrini*-like eggs from infected cats and dogs across various geographical localities in endemic areas of opisthorchiasis is needed, along with genotyping of *O. viverrini*-infected humans, to better understand their role as zoonotic parasites.

## 4. Materials and Methods

### 4.1. Sample Collections

**Figure 4:**
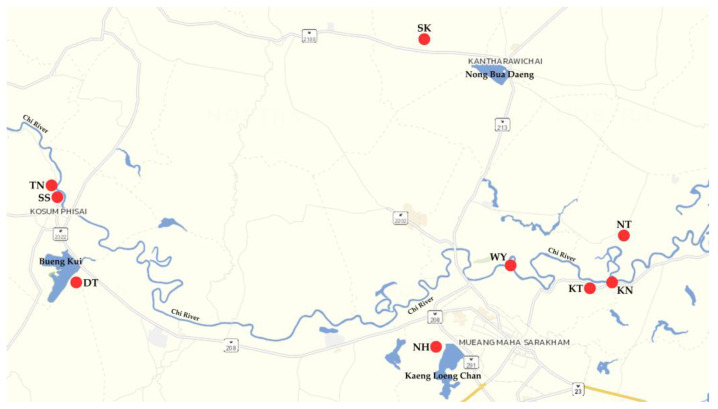
Map of the nine villages where the study sites are located in the Chi River Basin, Maha Sarakham Province, Thailand, as indicated by red dots. See Table 1 for more details on each village.

Fecal samples from 39 cats and 148 dogs were collected from nine villages between June and July 2022. Three of these villages and six others are located near natural reservoirs and the Chi River in Maha Sarakham Province, Thailand (Figure 4). A Patar NaCl enema was administered at a dosage of 20 mL for dogs and 10 mL for cats. The owners physically restrained the animals unless they were passive, in which case restraint was unnecessary. Once the enema was fully administered into the rectum, the restraint was loosened or released. Dogs were kept on a leash for the duration necessary for the enema to take effect, while cats were housed in cages until defecation was observed. After defecation, the fecal samples were collected from the ground or on newspapers in the cage using latex gloves and collection sticks to prevent cross-contamination and ensure sanitation. The fecal samples were placed in sterilized, labeled plastic bags and stored on ice until processing, which was conducted in the laboratory within 12 h. Any remaining fecal samples were aliquoted into 1.5 mL tubes and stored at −20 °C for direct fecal DNA extraction. The ages of cats and dogs ranged from 3 months to 10 years; however, their medical histories were unknown. All animals underwent a thorough external examination by veterinarians, and their health status was recorded.

### 4.2. Formalin-Ether Concentration Technique (FECT) Examination

Each fecal sample was examined for gastrointestinal (GI) nematode, cestode, and trematode eggs using a modified FECT, following a previously described report [30]. Microscopic examination was performed at 100×, with confirmation at 400× magnification when necessary. Parasite identification was based on established morphometric parameters, with particular emphasis on egg morphology and size [31]. All parasite-positive samples were re-examined twice to ensure accurate identification, and photographs were taken for documentation (Figure 1).

### 4.3. Molecular Analysis

Fecal samples that tested positive for *O. viverrini*-like eggs were extracted for DNA using the E.Z.N.A.^®^ Stool DNA extraction kit (Omega Bio-tek, Norcross, GA, USA), following the manufacturer’s protocol. In this study, intron 5 of domain 1 in the taurocyamine kinase (TkD1Int5) was used as a genetic marker. The amplification protocol and conditions for TkD1Int5 followed those described in a previous publication [21]. All PCR products were gel-purified using the E.Z.N.A.^®^ Gel Purification kit (Omega Bio-tek, Norcross, GA, USA). The purified PCR products were then cycle-sequenced through a commercial service provider (ATGC Co; Ltd., Bangkok, Thailand). Sequencing results were analyzed using Sequence Scanner ver. 1.0 to detect heterozygous patterns. Subsequently, the purified PCR product from a heterozygous sample was subjected to DNA cloning and plasmid sequencing, following the procedure outlined in a previous report [21]. Additionally, *O. viverrini* TkD1Int5 sequences from various localities in Thailand and Lao PDR, generated by a prior study [21], were also included in this analysis.

### 4.4. Data Analysis

The overall prevalence of intestinal parasites was estimated as the number of animals found to be positive for the presence of at least one species of parasite divided by the total number of animals examined. The prevalence of each parasite was calculated as the number of infected individuals over the total number of dogs/cats examined. The data were evaluated by descriptive statistics. All sequences generated in this study were aligned using ClustalW program version 1.4 [32] and optimized by sight in BioEdit program version 7.0.5.3 [33]. A minimum spanning haplotype network was constructed in the Network 5.0.0.3 program based on a median-joining network [34] using all sequences generated in this study.

## 5. Conclusions

This study highlights the high prevalence of parasitic helminth infections in outdoor cats and dogs near natural water sources in the Chi River Basin, Maha Sarakham Province, Thailand, supporting the hypothesis that these animals are potential sources of zoonotic infections. It also underscores the importance of genetic analysis in understanding the zoonotic potential of parasitic helminths in outdoor cats and dogs near natural water sources in the Chi River Basin, Maha Sarakham Province, Thailand. Notably, TkD1Int5 genotyping of *Opisthorchis viverrini*-like eggs from a cat revealed genetic similarity to *O. viverrini* populations previously identified in naturally infected cyprinid fish, supporting the hypothesis that cats play a significant role as reservoir hosts of this liver fluke. This finding aligns with previous studies indicating that the host specificity of human and cat genotypes is not distinct. These results demonstrate the critical role of molecular tools in elucidating transmission pathways and assessing the public health risks posed by parasitic helminths. Further genotyping of *O. viverrini*-like eggs from a larger sample size of infected cats, dogs, and humans across endemic regions is essential to clarify the genetic diversity, host specificity, and zoonotic transmission dynamics of these parasites.

## Figures and Tables

**Table 1:** Prevalence (%) of parasitic helminths in domestic cats and dogs from nine villages adjacent to Chi River and natural water sources in Maha Sarakham Province, Thailand.

Codes	Villages	Sub-District	District	Water Sources	Cat		Dog	
					*n*	No. Positive (%)	*n*	No. Positive (%)
KN	Keng Nuea	Koeng	Mueang	Chi River	4	1 (25)	16	8 (50)
KT	Keng Tai	Koeng	Mueang	Chi River	5	3 (60)	26	12 (46.2)
WY	Wang Yao	Koeng	Mueang	Chi River	5	5 (100)	14	7 (50)
NH	Non Hua Fai	Kaeng Loeng Chan	Mueang	Kaeng Loeng Chan *	0	0	16	5 (31.3)
TN	Tha Ngam	Hua Khwang	Kosum Phisai	Chi River	6	3 (50)	22	13 (59.1)
SS	Si Sook	Hua Khwang	Kosum Phisai	Chi River	10	9 (90)	15	8 (53.3)
DT	Don Tum	Lao	Kosum Phisai	Bueng Kui *	0	0	12	7 (58.3)
NT	Non Tan	Makha	Kantharawichai	Chi River	5	3 (60)	19	10 (52.6)
SK	Si Suk	Si Suk	Kantharawichai	Nong Bua Daeng *	4	3 (75)	8	6 (75)
				Total	39	25 (64.1)	148	76 (51.4)

* Reservoir; *n*, sample number.

**Table 2:** The prevalence of soil- and foodborne helminth infections in domestic cats and dogs.

Transmission Route	Genus	No. Positive (% Positive)	
		Cat (*n* = 39)	Dog (*n* = 148)
Foodborne	*Taenia*	0	16 (10.8)
	*Spirometra*	2 (5.1)	17 (11.5)
	*Hymenolepis*	1 (2.6)	3 (2.0)
	*Opisthorchis*	9 (23.1)	0
Soil-borne	*Ancylostoma*	18 (46.2)	55 (37.2)
	*Toxocara*	12 (30.8)	33 (22.3)
	*Strongyloides*	6 (15.4)	13 (8.8)
	*Trichuris*	0	3 (2.0)
	Total	25 (64.1)	76 (51.4)

*n*, sample size.

**Table 3:** Single and multiple parasitic infections in 76 infected dogs.

Type of Infection	No. of Species	Species Associated	Case (%)
Single infection	1 species (*n* = 32)	*Ancylostoma* sp.	19 (25.0)
		*Taenia* sp.	2 (2.6)
		*Toxocara* sp.	7 (9.2)
		*Spirometra* sp.	4 (5.3)
		Total	32 (42.1)
Multiple infections	2 species (*n* = 27)	*Ancylostoma* sp. + *Taenia* sp.	6 (7.9)
		*Ancylostoma* sp. + *Toxocara* sp.	8 (10.5)
		*Ancylostoma* sp. + *Spirometra* sp.	2 (2.6)
		*Ancylostoma* sp. + *Strongyloiodes* sp.	5 (6.6)
		*Taenia* sp. + *Spirometra* sp.	2 (2.6)
		*Taenia* sp. + *Toxocara* sp.	2 (2.6)
		*Toxocara* sp. + *Spirometra* sp.	1 (1.3)
		*Toxocara* sp. + *Hymenolepis* sp.	1 (1.3)
	3 species (*n* = 13)	*Ancylostoma* sp. + *Taenia* sp. + *Trichuris* sp.	1 (1.3)
		*Ancylostoma* sp. + *Taenia* sp. + *Hymenolepis* sp.	1 (1.3)
		*Ancylostoma* sp. + *Toxocara* sp. + *Spirometra* sp.	5 (6.6)
		*Ancylostoma* sp. + *Toxocara* sp. + *Strongyloiodes* sp.	4 (5.3)
		*Ancylostoma* sp. + *Strongyloiodes* sp. + *Trichuris* sp.	1 (1.3)
		*Taenia* sp. + *Toxocara* sp. + *Strongyloiodes* sp.	1 (1.3)
	4 species (*n* = 3)	*Ancylostoma* sp. *+ Taenia* sp. *+ Toxocara spp. + Strongyloiodes* sp.	1 (1.3)
		*Ancylostoma* sp. *+ Toxocara* sp. *+ Spirometra* sp. *+ Strongyloiodes* sp.	
		*Ancylostoma* sp. *+ Toxocara* sp. *+ Spirometra* sp. *+ Hymenolepis* sp.	1 (1.3)
	5 species (*n* = 1)	*Ancylostoma* sp.+ *Toxocara* sp. + *Spirometra* sp. + *Strongyloiodes* sp. + *Trichuris* sp.	1 (1.3)
		Total	44 (57.9)

*n*, sample size.

**Table 4:** Single and multiple parasitic infections in 25 infected cats.

Type of Infection	No. of Species	Species Associated	Case (%)
Single infection	1 species (*n* = 7)	*Ancylostoma* sp.	3 (7.7)
		*Toxocara* sp.	2 (5.1)
		*Opisthorchis* sp.	2 (5.1)
		Total	7 (17.9)
Multiple infections	2 species (*n* = 13)	*Ancylostoma* sp. + *Opisthorchis* sp.	1 (2.6)
		*Ancylostoma* sp. + *Toxocara* sp.	7 (17.9)
		*Ancylostoma* sp. + *Spirometra* sp.	1 (2.6)
		*Ancylostoma* sp. + *Strongyloiodes* sp.	2 (5.1)
		*Opisthorchis* sp. + *Toxocara* sp.	1 (2.6)
		*Toxocara* sp. + *Hymenolepis* sp.	1 (2.6)
	3 species (*n* = 5)	*Ancylostoma* sp. + *Opisthorchis* sp. + *Toxocara* sp.	1 (2.6)
		*Ancylostoma* sp. + *Opisthorchis* sp. + *Strongyloiodes* sp.	3 (7.7)
		*Opisthorchis* sp. + *Spirometra* sp. + *Strongyloiodes* sp.	1 (2.6)
		Total	18 (46.2)

*n*, sample size.

**Table 5:** Molecular diversity indices of *Opisthorchis viverrini* from different geographical localities in Thailand and Lao PDR and *O. viverrini*-like eggs from cats based on TkD1Int5 sequences.

Country	Codes	District/Province	Molecular Diversity Indices					
			*n*	S	H	Uh	Hd ± SD	Nd ± SD
Thailand	BR	Buri Ram	11	23	5	3	0.618 ± 0.164	0.0158 ± 0.0087
	KL	Mueang/Khon Kaen	12	37	12	9	1.000 ± 0.034	0.0309 ± 0.0164
	KB	Ban Phai/Khon Kaen	13	28	11	8	0.974 ± 0.039	0.0291 ± 0.0153
	KP	Phuviang/Khon Kaen	13	20	9	5	0.910 ± 0.068	0.0265 ± 0.0140
	CP	Chaiya Phum	10	28	10	7	1.000 ± 0.045	0.0360 ± 0.0194
	MD	Mukdahan	14	41	12	10	0.978 ± 0.035	0.0316 ± 0.0165
	MS	Maha Sarakham	10	9	9	6	0.978 ± 0.054	0.0038 ± 0.0024
	SKBg	Phang Khon/Sakon Nakhon	10	3	7	4	0.911 ± 0.077	0.0025 ± 0.0017
	SK	Mueang/Sakon Nakhon	11	14	8	5	0.927 ± 0.067	0.0093 ± 0.0053
	LP	Lampang	11	28	9	6	0.946 ± 0.066	0.0288 ± 0.0155
	CAT	Mueang/Maha Sarakham	10	23	8	8	0.933 ± 0.077	0.0393 ± 0.0212
Lao PDR	SV	Savannakhet	9	27	6	4	0.833 ± 0.127	0.0175 ± 0.0098
	KM	Khammouan	10	25	10	8	1.000 ± 0.045	0.0169 ± 0.0094
	NG	Nam Ngum/Vientiane	11	18	8	4	0.927 ± 0.067	0.0114 ± 0.0064
	TH	Tha Haue/Vientiane	8	19	8	6	1.000 ± 0.063	0.0152 ± 0.0087
	VT	Kam Pang Nakhon/Vientiane	11	15	7	4	0.873 ± 0.089	0.0103 ± 0.0058
	VV	Vang Viang/Vientiane	21	42	21	18	1.000 ± 0.015	0.0350 ± 0.0178
		Total	195	143	123	115	0.968 ± 0.007	0.0240 ± 0.0117

*n*, sample size; S, segregation site; H, number of haplotypes; Uh, unique haplotype; Hd, haplotype diversity; Nd, nucleotide diversity; and SD, standard deviation.

## Data Availability

All data are available upon request.
